# Tracking Progress on Person-Centered Care for Older Adults: Are We Doing Right by Racial and Ethnic Minorities?

**DOI:** 10.1093/geroni/igab046.2098

**Published:** 2021-12-17

**Authors:** Jane Tavares, Marc Cohen, Ann Hwang

**Affiliations:** 1 LeadingAge LTSS Center @UMass Boston, Boston, Massachusetts, United States; 2 University of Massachusetts Boston, University of Massachusetts Boston, Massachusetts, United States; 3 Commonwealth Care Alliance, Boston, Massachusetts, United States

## Abstract

Person-Centered care is integral and necessary to high-quality systems of care, providing a holistic approach and addressing the needs and preferences of individuals. Analyzing the 2014 and 2016 Health and Retirement Survey we measure the extent to which the health care system provides person-centered care, to whom and how its receipt affects satisfaction levels and service utilization. About one-third of individuals’ report that their preferences were only rarely or sometimes takes account. Results vary greatly by race, highlighting great disparities in person-centered care. One in four Hispanics and one in six Blacks report never having their preferences taken into account compared to roughly one in ten Whites. When people report that their preferences are ignored, they are more likely to forgo medical care and report lower satisfaction with the system. Strategies exist to strengthen and assure advancements in person-centered care, something particularly needed for people of color and low-income populations.

